# Disaster preparedness in hospices and specialized home palliative care services in Germany: a cross-sectional survey of team-leading nurses

**DOI:** 10.1186/s12912-026-04456-8

**Published:** 2026-02-20

**Authors:** Julia Ballmann, Michael Köhler, Sabine Pleschberger, Michael Ewers

**Affiliations:** 1 Institute of Health and Nursing Science, Charité – Universitätsmedizin Berlin, corporate member of Freie Universität Berlin and Humboldt-Universität zu Berlin, Augustenburger Platz 1, 13353 Berlin, Germany; 2 Medical University of Vienna, Center for Public Health, Vienna, Austria

**Keywords:** Hospice care, Disasters, Palliative care, Hospice and palliative care nursing, Terminal care, Disaster nursing

## Abstract

**Background:**

Hospices and specialized palliative home care services provide care for people with serious, life-threatening illnesses and those at the end of their lives. Disasters cause severe disruptions that exceed the capacity of structures and processes in a society. Given the increasing frequency and impact of disasters, it is crucial that these services maintain their functions for as long as possible, even under adverse conditions, to protect the people entrusted to their care. The aim of this research was therefore to examine whether and to what extent hospices and specialized palliative home care services in Germany are already prepared for disasters.

**Methods:**

We used an explorative, quantitative cross-sectional design. The intention was to conduct a full nationwide survey among nurses in team-leading roles in all hospices and specialized palliative home care services in Germany. A self-developed semi-standardized online questionnaire assessed perceived disaster awareness, organizational preparedness, and staff preparation. Descriptive statistics were used to analyze the survey data.

**Results:**

A total of 189 nurses in team-leading roles from all parts of Germany responded to the online survey (29% response rate). Overall, respondents considered disaster preparedness to be an important topic, but this has rarely been reflected in corresponding initiatives. 62% of respondents stated that their service does not have a business continuity plan, or that they were unaware of such a plan. An above-average share of respondents expressed the view that the nursing staff of these services should fulfil additional tasks before, during, and after disasters to ensure the safety of those in their care. Although nurses were generally considered competent to carry out these tasks, 70% of respondents agreed that further preparation for disasters was necessary.

**Conclusions:**

Our survey data suggest that hospices and specialized palliative home care services in Germany need to improve their disaster preparedness to ensure they can continue to provide specialized hospice and palliative care in times of adversity. This requires organizational initiatives in collaboration with other healthcare and civil protection services, as well as personnel development measures. Nurses in leadership positions could play a key role in this.

**Supplementary Information:**

The online version contains supplementary material available at 10.1186/s12912-026-04456-8.

## Background

Inpatient hospices and specialized palliative home care services are usually small-scale, community-based organizations that provide skilled and comprehensive care for people with serious, life-threatening illnesses and those at the end of their lives, most of whom have complex and extensive support needs [1]. The main goal of these specialist care services is to ease the suffering of severely ill people, improve their quality of life, and ensure they are comfortable, safe, and treated with dignity [2]. In doing so, these multiprofessional services focus not only on the severely ill or dying person, but also on their relatives and other informal carers.

Internationally, there is a growing demand for these kinds of specialized care services due to far-reaching demographic and epidemiological changes [3, 4]. In Germany, this was countered by a considerable expansion of hospice and palliative care services during the last two decades in all health and social care settings and on different levels of care [5]. By 2023, around 350 palliative care units in hospitals and 286 inpatient hospices were established nationwide [5]. In accordance with the legal requirements for operation and funding, inpatient hospices usually offer between 8 and 16 beds to ensure a sense of familiarity [6]. In addition, 403 specialized palliative home care services (in German: *S*pezialisierte *a*mbulante *P*alliativ*v*ersorgung, SAPV) were established throughout the country [5]. Based on German legislation, these small-scale, team-based services provide skilled hospice and palliative care (HPC) in private and elderly homes, funded by the statutory health and long-term care insurance to allow severely ill and dying patients to stay in their living environment. Furthermore, several palliative care advisory teams have been set up to support regular care providers (general practitioners, home nursing care services, nursing homes) in providing general hospice, palliative, and end-of-life care in the community.

Nurses form the backbone of the multiprofessional teams providing these health and social care services. Based on quality agreements with the health and long-term care insurance, inpatient hospices must regularly employ 12 full-time licensed practical nurses [6]. In addition to licensed practical nurses, less qualified carers, therapists, social workers, housekeeping, and administrative staff, a palliative care physician must always be available in inpatient hospices [7]. In this context, it should be noted that nurses in Germany are still predominantly educated in three-year vocational programs, mostly provided by hospital-based nursing schools [8]. If these nurses wish to work in this special field, they usually need to prove that they have completed further theoretical and practical training in HPC with a scope of 160 h. Based on quality agreements, approximately 50% of all nurses employed in inpatient hospices should have this additional qualification; it is mandatory for team-leading nurses. SAPV teams are also relatively small; they usually consist of palliative care physicians and hospice and palliative care nurses. SAPV teams regularly have a minimum of 4 full-time positions for licensed practical nurses with further training in HPC [9]. Team-leading nurses in hospices and SAPV teams are responsible for the organization and continuity of health and social care, the quality of the medical and nursing care services provided by these organizations, and for the safety of the patients and their relatives.

Safety deserves special attention, as inpatient hospices and specialized palliative home care services, like other health and social organizations, are increasingly confronted with a growing risk of crises and disasters, even in Germany [10]. The United Nations Office for Disaster Risk Reduction (UNDRR) defines disasters as events that cause far-reaching disruptions in a community or society that exceed the capacity of its structures and processes [11]. There is a wide range of hazards that can lead to disasters, including extreme weather events, technical failures, violent conflicts, and CBRNE events, as well as the associated cascading effects, depending on the level of exposure, vulnerability, and resources. Therefore, an all-hazard approach should be followed to prepare for all kinds of disasters. The far-reaching consequences of disasters, particularly for people with complex health problems and specific support needs, were only recently revealed by the COVID-19 pandemic. This public health crisis highlighted systemic weaknesses and gaps across all sectors of the health and social care system, including HPC services [12]. The COVID-19 pandemic led, for example, to dehumanization of care and to a lack of resources, information and expertise in HPC services [12]. Due to their special situation and complex needs, people cared for by these services were among the high-risk populations during this specific health crisis [12, 13]. It has become clear that if hospices and palliative care services do not take the necessary precautions to protect those in their care and provide skilled services continuously, even under adverse circumstances, there is a high risk of significant physical and mental health problems, and even premature death [14, 15].

The experience of the COVID-19 pandemic is certainly one reason that the importance of disaster preparedness in all areas of health and social care, especially in settings where particularly at-risk populations are cared for, has been increasingly emphasized in the recent past [16, 17]. However, other experiences, including the consequences of climate change and geopolitical disruptions, have also made it clear that better preparedness for unusual events is urgently needed to increase resilience in health and social care. Every healthcare service, even small-scale decentralized ones, should be able to continue operating for as long as possible in the event of a disaster to protect the lives and health of those in their care and reduce the strain on critical infrastructure. This can be achieved through proactive, tailored disaster preparedness initiatives [18], which include, for example, organizational measures such as the development of business continuity plans, risk management strategies, emergency response plans for employees, collaboration with other healthcare providers, evacuation plans, or personnel development measures such as skills development, employee training, and drills with first responders and civil defense organizations [19, 20].

However, little is known about the extent to which inpatient hospices and specialized palliative home care services are already aware of the disaster risks that could affect them in the future, particularly in Germany, which has largely been spared major exceptional events in the past. It is also unclear what disaster preparedness measures these services have already put in place or how their team-leading nurses perceive the tasks and responsibilities of licensed nurses before, during, and after disasters, as well as their competencies in the field of emergency and disaster nursing. With our research, we intended to address these research gaps. We aimed to explore disaster awareness and preparedness among inpatient hospices and SAPV services in Germany, and based on these findings, to strengthen the resilience of these indispensable services for one of the most at-risk populations in the health and social care sector.

## Methods

### Design and ethics

We conducted an exploratory cross-sectional study using a self-developed semi-standardized online survey among team-leading nurses in inpatient hospices and specialized palliative home care services. This study was conducted in full compliance with the ethical principles set out in the Declaration of Helsinki for medical research involving human subjects, and was approved by the Charité – Universitätsmedizin Berlin Ethics Committee as part of the CRISPALL project (EA2/258/24).

### Recruitment and setting

The online survey was conducted in Germany between mid-January and mid-March 2025. Hospices and home care services were identified through cooperation with the two most important German hospice and palliative care organizations (Deutsche Gesellschaft für Palliativmedizin e.V. and Deutscher Hospiz- und PalliativVerband e.V.). These organizations work closely together and operate a joint web-based platform on which, by December 2024, all inpatient hospices and 93% of all SAPV services in Germany were listed with their contact details [5]. The platform was updated by the associations a month before the start of this study. Using these contact details, we were able to reach 657 of 664 listed services. Invitations to participate in the survey were sent by email to the team-leading and responsible nurses of these services in mid-January 2025. In cases where no direct email address for these individuals was available, we requested that the invitation be forwarded to the appropriate person, using an organizational email address. Participants were asked to provide written consent and were informed that their participation was entirely voluntary and could be withdrawn at any time without consequences. To increase participation, reminders were sent by email two and four weeks after the initial invitation to participate.

### Data collection

As there is no standardized instrument for assessing the disaster preparedness of inpatient hospices and specialized palliative home care services, either in Germany or internationally, we have invested considerable effort in a self-developed questionnaire: In several discussion rounds, two authors (JB, ME) compiled and analyzed information from relevant disaster-related literature [exem. 12, 13, 21] using it to define domains and items for the online questionnaire. The domains and items were then reviewed for comprehensibility and usability by another member of the research team (MK), after which they were either included or excluded by consensus. Responses were mostly given on five-point Likert scales, but also nominal scales with ‘yes’, ‘no’, ‘don’t know’, or ‘neither’ were used. A pilot test was conducted with 12 HPC representatives and 9 disaster nursing stakeholders, with the main goal of accurate wording and comprehension. The instrument was then further optimized based on feedback from this process. The final semi-standardized questionnaire was distributed via the platform SoSciSurvey [22]. Team-leading nurses from hospices and from SAPV teams received the same questionnaire. The data were analyzed descriptively with SPSS version 30. Datasets with more than 50% missing data or lacking informed consent were excluded prior to analysis. Analyses were conducted on an itemwise basis. Each item was analyzed based on all available valid responses, resulting in varying sample sizes across items. No composite variables, indices, or subscales were calculated. As the questionnaire was designed for exploratory, content-oriented assessment rather than for measuring latent constructs, no psychometric testing was performed. Responses to the open-ended questions were used to further illustrate the results.

The final instrument in German language covered four domains, additional literature sources (for further development references see appendix 1):

1. Personal and professional background.

Respondents were asked to provide information about their background (demographics, qualifications) as well as some key information about the services in which they were active.

2. General disaster awareness.

Disaster awareness can be defined as “the extent of knowledge about disaster risks and the factors that lead to disasters” [23; p. 1]. This knowledge has a significant influence on the measures taken to prepare for disasters and to prevent serious impacts. In this study, the disaster awareness of the participants was assessed through two factors: They were asked to estimate the perceived probability of occurrence for different types of disasters and the perceived impact of a disaster on their service from their point of view. Both factors were rated on a 3-point scale (low, medium, high). Following the classifications of the WHO and the UK Health Protection Agency [24], we categorized disasters into four types: (1) natural hazards (flood, storm, forest fire), (2) biological hazards (epidemics, pandemics), (3) technological hazards (cyber-attack, nuclear or chemical accident), (4) social hazards (terrorism, war, rampage).

3. Organizational disaster-preparatory measures.

Disaster preparedness, as understood by the European Union, is “a set of measures undertaken in advance by governments, organizations, communities, or individuals to better respond to and cope with the immediate aftermath of a disaster” [25]. We therefore asked team-leading nurses if and how they had already developed such preparatory measures in their organization (e.g., having a business continuity plan, preparation for evacuation, connecting with security services like firefighters or emergency relief organizations).

4. Disaster preparation of nursing staff.

The preparedness of licensed practical nurses in HPC regarding crises and disasters was another domain of the instrument. Personnel preparedness also plays an important role when it comes to promoting resilience to disasters and averting potential damage caused by hazardous events. The questions were therefore posed as to what specific disaster-related preparation tasks team-leading nurses see for their staff, what competencies they already have for these tasks, and if there are further educational needs. The starting point for the development of this part of the questionnaire was the German version [26] of the International Council of Nurses (ICN) Core Competencies for Disaster Nursing [21].

## Results

### Sample

After cleaning out data sets with more than 50% missing values, 189 team-leading nurses of inpatient hospices or specialized palliative home care services participated in the survey, corresponding to a response rate of 28.8%. The response rates were comparable between inpatient hospices (30.1% response rate; *n* = 86) and SAPV services (27.0% response rate; *n* = 100). The characteristics of the participants are shown in Table 1. Most services focused on adults as their patient group (*n* = 146, 77.2%), while 10.6% (*n* = 20) focused on children/youth, and 3.2% services (*n* = 6) treated both age groups. Organizations from all 16 federal states of Germany were reporting back, with the highest response rate of the state of Saarland (55%) and the lowest from all services in Thuringia (11%). The hospice services were founded between 1986 and 2021 and have existed for an average of 19 years. In comparison, SAPV services were founded between 2003 and 2023 and have existed for an average of 12 years. Most hospices reported having between 8 and 12 beds (*n* = 62; 77%), in accordance with legal regulations enacted in 2010. Respondents reported an average of 17.4 full-time nursing positions for hospices and 8.1 for SAPV services. Due to the limited number of beds available in hospices and the relatively small number of staff, both hospices and SAPV services are categorized as small-scale healthcare organizations.

**Table 1:** Characteristics of team-leading nurses

Survey characteristics of team-leading nurses of inpatient hospices and SAPV services	
Gender (*n* = 186):	78% female, 20% male, 2% not specified
Age (*n* = 184):	M = 50,6 years (SD = 9.3)
Active in Civil and Disaster Protection (*n* = 186):	currently: 5/189 (2.6%) used to: 13/189 (6.9%)
Work experience (*n* = 168):	M = 24.8 years as a nurse (SD = 9.7) M = 11.7 as a nurse in HPC (SD = 6.6) M = 10.2 as a team-leading nurse (SD = 7.2)
Higher qualification (*n* = 189):	Bachelor’s degree (14.8%) Master’s degree (11.6%) Diploma (equivalent in level to a Master’s degree, 7.4%) Doctorate (2.6%)

### General disaster awareness

**Fig. 1:**
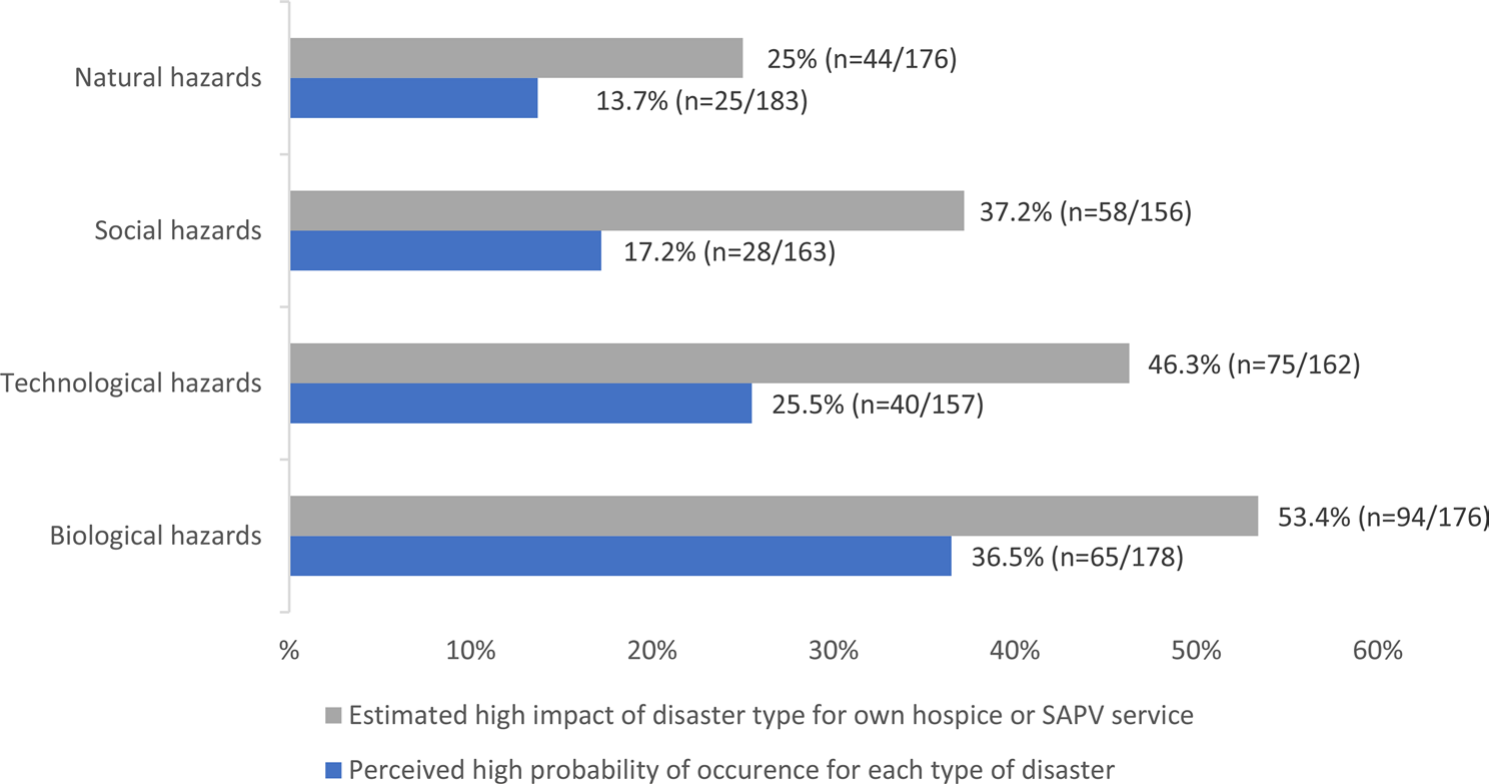
Chosen high probability or high impact across disaster types

Survey findings indicated rather low levels of disaster probabilities in hospices and SAPV service, while perceived possible impacts of disasters were rated higher, as shown in Fig. 1, where only high probability or high impact is presented. Biological hazards were perceived as the most critical threat to HPC services combining both high probability and high impact. A high probability was reported by 36.5% (*n* = 65/178; medium 52.8%; low 10.7%), while 53.4% (*n* = 94/176) rated the impact as high (medium 36.9%; low 9.7%). Natural hazards were consistently rated as least relevant. Only 13.7% (*n* = 25/183) assessed their probability as high (medium 44.3; low 42.1%), and 25% (*n* = 44/176) perceived a high impact on services, whereas 42.6% rated the impact as low (medium 32.4%). Technological hazards occupied an intermediate position. A high probability was indicated by 25.5% (*n* = 40/157; medium 49%, low 25,5%), alongside a comparatively high perceived impact, with 46,3% (*n* = 75/162) rating it as high (medium 37%, low 16.7%). Social hazards showed moderate probability and impact assessments. High probability was reported by 17.2% (*n* = 28/163; medium 46.6%, low 36.2%), while 37.2% (*n* = 58/156) rated the impact as high (medium 34.6%, low 28.2%).The majority of participants (*n* = 161) reported that their services had been affected by a pandemic (presumably the COVID-19 pandemic). In addition, 27 out of 189 participants already experienced other disasters, including floods, power losses, evacuations due to bomb defusing, fires, storms, and cyber-attacks.

At the same time, the team-leading nurses participating in this survey perceived disaster preparation as an important topic: on a 10-point Likert scale they were asked how important disaster preparation is for HPC services in Germany (from 1 = unimportant to 10 = very important). 119 team-leading nurses rated the importance of disaster preparedness with a mean of 7.6 (SD = 2.2; median value = 8), with no significant difference between inpatient hospices or SAPV services.

Part of the questionnaire was about rating different areas concerning their own service, which were affected by the COVID-19 pandemic (see Table 2). On a scale from 1 (‘did not influence at all’) to 4 (‘strongly influenced’), the greatest reported impacts were the absence of volunteers (M = 3.4), change in team spirit (M = 3.4), additional effort in educational support (M = 3.3), and difficulties in interdisciplinary work (M = 3.3). The two least influential items were increased patient load (M = 2.4) and less time for patients and relatives (M = 2.6). Participants were also asked whether they believe the COVID-19 pandemic has contributed to improved preparedness for future disasters in HPC. While 27% (*n* = 51/187) indicated a perceived improvement, 31% (*n* = 59/187) did not, and 41% (*n* = 77/187) were unsure.

**Table 2:** Impact of the COVID-19 Pandemic on HPC services

	Impact of the COVID-19 Pandemic Mean, Standard Deviation & Median*
Absence of volunteer staff	M = 3.4, SD 1.0; Mdn = 4; (*n* = 173/189)
Changed team spirit	M = 3.4, SD 0.9; Mdn = 4; (*n* = 173/189)
Additional effort in educational support	M = 3.3, SD 0.8; Mdn = 4; (*n* = 182/189)
Difficulties in interdisciplinary work	M = 3.3, SD 0.9; Mdn = 4; (*n* = 184/189)
Difficulties in grief support	M = 3.3, SD 1.0; Mdn = 4; (*n* = 183/189)
Difficulties in final phase	M = 3.2, SD 0.9; Mdn = 3.5; (*n* = 184/189)
Additional effort in therapeutic interventions	M = 3.1, SD 1.0; Mdn = 3; (*n* = 184/189)
Additional effort in symptom management	M = 2.8, SD 1.0; Mdn = 3; (*n* = 185/189)
Less time for patients and relatives	M = 2.6, SD 1.0; Mdn = 3; (*n* = 181/189)
Increased patient load	M = 2.4, SD 1.0; Mdn = 2; (*n* = 179/189)

*1 (did not influence at all) to 5 (strongly influenced)

When asked to provide an estimation of whether or not HPC patients need more support than other patients when confronted with a disaster, team-leading nurses reported relatively balanced responses on a 5-point Likert scale. Specifically, 30% (*n* = 52/176) of respondents indicated that they did not anticipate a need for increased support, while 28% (*n* = 49) remained undecided. Conversely, 43% (*n* = 75) of nurses expressed the view that their patients would need more support than others. When asked the same question for relatives and families, 64% (*n* = 112/176) of the participants reported a greater need of support for relatives and families of HPC patients than other relatives in disasters, 18% (*n* = 31) opposed this statement, and 19% (*n* = 33) were undecided.

### Organizational disaster-preparatory measures

In this study, we primarily asked for organizational disaster preparatory measures: 65/170 (38%) services had a business continuity plan, while 68 (40%) had none, and 37 (36%) team-leading nurses did not know if they had one. Of the 65 services that had a disaster concept, less than half (*n* = 30, 46%) had it agreed upon their local hazard prevention authority.

We also asked about the local authorities and institutions the nurses already had contacted regarding disaster preparation: While most HPC services did not have contact to any, some (*n* = 54/171; 32%) reported that they had talked to the regional fire department or the federal agency for technical relief (THW), a civil protection organization of Germany providing technical and humanitarian assistance in disasters. Other rescue and emergency services were contacted from 43 (25%) HPC services, and some also reached out to security agencies, such as the police and military (*n* = 34, 20%). Some nurses had also contacted the local council or the local health authority.

Three-quarters of HPC services (*n* = 127/170; 75%) had not nominated a person responsible for disaster preparation. In 28 cases (17%), specific persons were named for this task, 20 of whom (71%) were nurses. At the same time, a third of respondents (*n* = 55/168, 33%) stated that as nurses in leadership positions, they were officially responsible for disaster preparedness in their organization. Almost two-thirds (*n* = 100, 60%), on the other hand, said that this was not the case for them. Focusing on the services that did not have a designated person for disaster preparation, also the nurse in the leadership role did not see this as his or her responsibility (*n* = 74, 59%), while 32% (*n* = 40) did consider it to be their responsibility.

### Disaster preparation of nursing staff

Participants were asked to rate selected tasks that licensed practical nurses in HPC should be able to perform before, during, and after disasters based on the ICN Core Competencies – particularly those at the first level of this framework for all nurses [21].

As presented in Table 3, the participants considered ‘Maintaining safety for oneself and other colleagues’ to be the most important task, while ‘Preparing patients and their relatives in advance’ was considered least important. We also asked whether the licensed practical nurses in HPC currently have sufficient competencies to perform these tasks. Overall, the ratings are lower than the importance score and show greater dispersion as reflected in higher standard deviations.

**Table 3:** Tasks nurses should perform and the competency to do so

Task	Perception of tasks nurses should perform before, during, and after disasters; Mean, Standard Deviation & Median*	Perception of competency for Task; Mean, Standard Deviation & Median*
Maintaining safety for oneself and other colleagues	M = 4.6; SD 0.6; Mdn = 5; (*n* = 174/189)	M = 4.0; SD 0.8; Mdn = 4; (*n* = 174/189)
Conveying safety to patients and their relatives in case of disaster	M = 4.5; SD 0.6; Mdn = 4.5; (*n* = 176/189)	M = 3.9; SD 0.8; Mdn = 4; (*n* = 173/189)
Representing the interests of their patients vis-à-vis third parties (e.g., emergency services)	M = 4.2; SD 0.8; Mdn = 4; (*n* = 173/189)	M = 3.8; SD 0.9; Mdn = 4; (*n* = 171/189)
Providing HPC where patients are located, e.g., in a care facility or shelter	M = 4.1; SD 0.9; Mdn = 4; (*n* = 173/189)	M = 3.9; SD 1.0; Mdn = 4; (*n* = 168/189)
Preparing patients and their relatives in advance	M = 3.8; SD 0.8; Mdn = 4; (*n* = 171/189)	M = 3.2; SD 0.9; Mdn = 3; (*n* = 173/189)
Adapt established standards to the respective circumstances in the event of a disaster		M = 4.0; SD 0.8; Mdn = 4; (*n* = 173/189)

*1 (don’t agree at all) to 5 (totally agree)

In general, most nurses in leadership roles emphasized the need for additional training in disaster preparedness for licensed practical nurses working in HPC: On a scale from 1 (‘Do not agree at all’) to 5 (‘Fully agree’), 12% (*n* = 20/166) of the respondents disagreed, while 19% (*n* = 33) indicated ‘neither’. 70% (*n* = 122) agreed to the need for further disaster preparedness training for licensed practical nurses employed in their inpatient hospices or SAPV services.

Taken together, these findings provide a first overview of current perceptions of disaster preparedness in hospices and SAPV services, which will be discussed in relation to existing literature and contextual factors in the following section.

## Discussion

The aim of this study was to explore the perceived disaster preparedness of HPC services in Germany from the perspective of team-leading nurses. The findings reveal that while these nurses emphasized the importance of disaster preparedness, they identified major shortcomings, such as inconsistent structural frameworks, insufficient training of staff, unclear responsibilities, and a lack of strategies to improve disaster preparedness of their small-scale healthcare services.

The findings underscore the need to expand disaster awareness and preparedness in HPC, moving beyond a pandemic-centered perspective towards a comprehensive, all-hazard approach. The growing number of people in need of community-based HPC, together with an increasing likelihood of disasters with their devastating consequences, calls for greater disaster awareness and improved preparedness [10]. In contrast to hospitals – where preparedness for exceptional events is mandatory in many countries, and best practices are often shared [17] – the long-term care sector [27], and, in particular, the HPC sector has long been overlooked. Yet these services care for highly vulnerable patients whose dependence on continuous and specialized care leaves them at considerable risk during and after disasters [14]. Although some disaster preparatory measures have been implemented in HPC in response to the COVID-19 pandemic, they remain narrow in scope [12, 28]. This was also evident in our survey: biological hazards, including pandemics, –were consistently rated as most likely and most impactful. This emphasis may be influenced by recent experiences with COVID-19 and may be explained by the fact that only 14% of HPC services in our survey stated that they had already been affected by non-pandemic-related disasters such as floods or major cyber-attacks. Even though international disaster databases indicate that in many regions extreme weather events currently account for a substantial share of health-related disaster [29]. A COVID-19-related bias is also visible in research literature, which shows numerous publications concerning HPC in the context of COVID-19 [12, 13], while other hazards were underrepresented, especially natural disasters [30]. The finding that only 27% of team-leading nurses believed that the HPC sector is better prepared for disasters after the COVID-19 pandemic might explain that they have acknowledged, that effective disaster preparedness requires differentiated measures for a broad range of hazards and that preparation needs to increase in line with an all-hazard approach. Although an all-hazards approach has not yet been widely adopted in HPC, its integration [15] – for example, through the inclusion of geological and environmental data provided by authorities responsible for hazard prevention –promises high potential to enable a more accurate assessment of actual risks [13, 31].

Effective disaster preparedness in HPC requires proactive planning and networking among local services and stakeholders. Cooperation and collaboration can enhance preparedness and improve responses to adverse events through better understanding and coordination [20, 32, 33]. Our findings point to structural deficits in organizational preparedness of HPC services, including a lack of concepts, unclear responsibility structures, and, in particular, missing external collaboration. More than half of the surveyed services in Germany lacked a business continuity plan, and three-quarters had no designated person responsible for disaster preparedness. Our findings align with similarly low levels of preparedness observed in other home healthcare services [34]. Structural factors such as limited resources, absence of regulatory requirements, and the small size of organizations are barriers to development of preparedness plans. Although considered an important preparatory measure, developing, implementing, and testing a business continuity plan can be overwhelming for a small service provider [34]. In such cases, establishing cooperation and network structures could be helpful. Literature indicates that small home-visit nursing operators collaborated to develop a shared plan for all services in their area, which individual services could adapt for themselves [33, 35]. In Germany, publicly funded support measures exist to enable HPC providers to network with each other at the community level [36], but so far, these collaborations have rarely included disaster preparation measures. Although these collaboration initiatives are valuable for building mutual understanding and coordination among the HPC providers and their partners, adding other health care providers and, in particular, disaster preparedness, disaster relief, and emergency organizations to such networks would mark a major step toward better disaster preparedness. Without prior collaboration, logistical obstacles such as limited access to medicines or care supplies during disasters are more difficult to overcome [30]. In addition, such networks can contribute to building trusting relationships between health care organizations, emergency services and disaster response teams, which is particularly important in times of crisis and disaster.

Next to these organizational dimensions, our results highlight the crucial role of team-leading nurses for disaster preparedness in HPC. This is particularly relevant when it comes to the challenges in human resources development, such as the targeted preparation of the staff working in HPC for their tasks before, during, and after disasters, as well as the staff planning in the event of a disaster. In our survey, team leading nurses were able to identify some specific tasks for nurses before, during, and after disasters, and they expressed confidence that their staff would be able to handle them properly. For example providing safety to oneself and other staff members was continuously identified as a relevant nursing task, in line with other studies [37]. At the same time, however, the team leading nurses in our survey identified a considerable need for further training when it comes to coping with care tasks in the context of disasters. This aligns with studies indicating that healthcare workers generally exhibit inadequate disaster preparedness across most countries [38], although regional differences exist, with high-income countries reporting a higher level of perceived preparedness and less disaster-related fatalities [39, 40]. Regional differences also exist in the basic education of nurses, which has an impact on their operational readiness in the event of a crisis or disaster. In this context, it is important to remember that most nurses in Germany are educated through a three-year vocational training at secondary level – in contrast to most countries around the world, where an academic degree is required to qualify for the profession. Even if nurses in HPC in Germany have an additional 160 h-palliative-care-training, formal disaster-preparedness falls short. However, this is not unique to Germany, because although the ICN emphasizes that disaster preparation needs to be a standard part of nursing practice, to maintain safety for oneself, colleagues, and others [21], this requirement is not consistently met. This shortcoming is echoed by research which highlights the need of continuous training and drills [14, 41, 42] to ensure sufficient operational readiness in the event of an incident. Accordingly, the participants in our study call for more disaster-specific training for their staff in HPC. Not least, this reflects the special responsibility of team leading nurses in HPC to ensure that all their colleagues and employees have the necessary skills to protect themselves and the people entrusted to their care in the event of an incident [43]. However, in order to fulfil this responsibility, they will not only need an awareness of the problem, but also appropriate resources, organizational and political support.

### Limitations

A strength of our research is that, to our knowledge, it is the first nationwide survey in Germany on disaster preparedness in HPC. While it provides valuable insights from team-leading nurses, several limitations should be considered: Hospices and SAPV services were identified through an online registry maintained by German palliative care associations, which allowed to reach most services. However, since the registry is not mandatory, we have missed about 7% of the SAPV teams. It is possible that, particularly among SAPV teams, invitations were not sent directly to the nurses due to functional emails. Nevertheless, with a 29% response rate, results were within the expected range for a nationwide online survey with direct recruitment via mail [44]. Given the exploratory nature of the study, a self-developed instrument was used, which was neither standardized nor validated. We addressed this point by developing the instrument closely on available scientific literature and conducting a pretest with experts from both HPC and disaster nursing. Aa a further limitation availability heuristic has to be considered, whereby the heightened salience of recent experiences with the COVID-19 pandemic might have contributed to how importance and impact of disasters were rated as well as how interpretations were formed. Furthermore, we assessed perceived preparedness and self-reported competencies of team-leading nurses, which may not fully reflect actual preparedness, or performance. With the national focus on Germany, transferability of results is limited, as structural, educational, and political contexts vary internationally. Nevertheless, these findings can serve as a starting point, providing an initial overview of perceived disaster preparedness in the nursing context and underscoring the need for further research into nursing roles, tasks, and preparedness in greater depth.

## Conclusions

This national survey provides much-needed baseline data for improving disaster preparedness in German HPC settings. Within this sample, team-leading nurses reported notable gaps in formal disaster preparedness structures (e.g. absence of business continuity plans and designated responsibilities) and a primary focus on biological hazards. These findings suggest that disaster planning in many German hospice and SAPV services does not yet consistently follow an all-hazards approach. Our findings raise concerns and highlight the relevance of organizational and personnel capacity development policies, including clearly defined responsibilities, disaster-focused training for nurses, and institutional support for team-leading nurses. Improvements in embedding disaster response systems and building networks with disaster relief organizations and other healthcare services may also enhance care continuity during adverse events. Further, legislative policy mandating disaster preparedness equivalent to that required for hospitals and general home healthcare in Germany could improve awareness and preparation. Finally, more evidence-based research is needed to identify interventions to strengthen HPC services, disaster competencies of nurses and nursing leadership in disaster preparedness.

## Supplementary Information

Below is the link to the electronic supplementary material.

Supplementary Material 1

## Data Availability

The dataset used and analyzed during the current study are available from the corresponding author on reasonable request.
