# Supplementary material for: Disaster preparedness in hospices and specialized home palliative care services in Germany: a cross-sectional survey of team-leading nurses
Source: BMC Nurs. 2026 Feb 20;25:285. doi: 10.1186/s12912-026-04456-8 (PMC13032588; doi:10.1186/s12912-026-04456-8)
Supplement: Supplementary file 1 — Supplementary Material 1 [file 12912_2026_4456_MOESM1_ESM.docx]

Appendix 1: Development of Survey Instrument for Disaster Preparedness in Hospices and SAPV-Teams

| Part of Questionnaire | Number of Items | Indicators | Based on following Literature |
| --- | --- | --- | --- |
| Personal and professional background | - 15 | 1. Gender, 2. Year of birth, 3. Qualification, 4. Experience as nurse, 5. Experience in HPC, 6. Experience in team-leading positions, 7. (previously) active in disaster relief organizations, 8. Setting, 9. Full- or Part-time, 10. Just leading position or also clinical care, 11. Facility Size, 12. Number of Nursing Staff, 13. Federal county, 14. Age group of patients, 15. Year of Establishment. | - Deutsche Gesellschaft für Palliativmedizin (DGP), 2024: Wegweiser Hospiz- und Palliativversorgung Deutschland, <https://www.wegweiser-hospiz-palliativmedizin.de/> |
| General disaster awareness | - 10 | 1. Probability of occurrence of natural hazard; 2. – of biological hazard, 3. - of technological hazard, 4. – of social hazard. 5. Impact of natural hazard, 6. – of biological hazard, 7. - of technological hazard, 8. – of social hazard. 9. Impacted by COVID-19, 10. Impacted by further disasters so far. | - WHO, UK Health Protection Agency and partners, 2011: Disaster Risk Management for Health, <https://cdn.who.int/media/docs/default-source/documents/disaster-risk-management-for-health.pdf> - Deutsche Gesellschaft für Technische Zusammenarbeit (GTZ) GmbH, 2004: Guidelines Risk Analysis – a Basis for Disaster Risk Management, <https://www.careemergencytoolkit.org/wp-content/uploads/2017/03/34_9.pdf> - Patel RK, Pamidimukkala A, Kermanshachi S, Etminani-Ghasrodashti R: Disaster Preparedness and Awareness among University Students: A Structural Equation Analysis. International Journal of Environmental Research and Public Health 2023, 20(5):4447. |
|  | - 11 | 1. Influence of Covid-19 on Disaster Preparedness: Increased patient volume, 2. Absence of volunteers, 3. Less time for patients, 4. Differences in team spirit, 5. Additional efforts with symptom control, 6. Additional efforts with therapeutic measures, 7. Additional efforts with educational tasks, 8. Difficulties in providing support during the final phase, 9. Difficulties in grieving, 10. Difficulties concerning interprofessional work, 11. Better prepared after COVID-19. | - Plagg et al., 2023: Hospice and Palliative Care during Disasters: A Systematic Review - Bausewein et al., 2022: National strategy for palliative care of severely ill and dying people and their relatives in pandemics (PallPan) in Germany – study protocol of a mixed-methods project [Supplementary files for online surveys of pandemic teams and inpatient hospices] - Bradshaw et al., 2021: Understanding and addressing challenges for advance care planning in the Covid-19 pandemic: An analysis of the UK CovPall survey data from specialist palliative care services |
| Organizational Disaster Preparatory measures | - 14 | 1. More support needed for HPC patients, 2. More support needed for relatives in HPC, 3. Legislation requirement, 4. Business continuity plan available, 5. Responsible person for disaster preparedness, 6. Responsible person is team-leading person, 7. Contact with emergency services and disaster control, 8. Preparation for power and water outages, 9. Preparation for Evacuation, 10. Plans for continued care for our patients, 11. Plans for own personnel to continue care in other locations, 12. Possibility to increase workforce with volunteers, 13. Possibility to admit additional patients, 14. Importance of disaster preparedness for HPC and own organization. | - WHO, 2019: Health Emergency and Disaster Risk Management Framework, <https://iris.who.int/bitstream/handle/10665/326106/9789241516181-eng.pdf> - Lessinnes et al., 2023: Evidence of Disaster Planning by Home Care Providers: An Integrative Literature Review - Segey et al., 2024: Nursing roles in disaster zones: Experiences and lessons from Turkey’s 2023 earthquakes - Thompson et al., 2024: Palliative care emergencies in a hospice setting: Using simulation-based training to improve nursing confidence - Baker Rogers, 2023: Hospices and Emergency Preparedness Planning: A Scoping Review of the Literature - European Union, 2023: Disaster preparedness [<https://civil-protection-humanitarian-aid.ec.europa.eu/what/humanitarian-aid/disaster-preparedness_en>] |
| Disaster preparedness of nursing staff | - 12 | 1. Tasks of nurses: Prepare patients and relatives, 2. Conveying safety, 3. Maintaining safety for oneself and other colleagues, 4. Representing patients’ interests vis-à-vis third parties, 5. Providing HPC where patients are located, 6. Competency for tasks: Prepare patients and relatives, 7. Conveying safety, 8. Maintaining safety for oneself and other colleagues, 9. Representing patients’ interests vis-à-vis third parties, 10. Providing HPC where patients are located, 11. Adapt established standards to the circumstances, 12. Need for further training. | - ICN, 2019: Core Competencies in Disaster Nursing Version 2.0) - Ewers & Köhler, 2024: Consensus based adaption of the ICN Core Competencies in Disaster Nursing for German-speaking countries (CORE) - Kelly et al., 2023: “I can’t make all this work.” End of life care provision in natural disasters: a qualitative study - Wong et al., 2023: The critical role of nurses and midwives in disasters |
